# Atomic Layer Deposition for Preparing Isolated Co Sites on SiO_2_ for Ethane Dehydrogenation Catalysis

**DOI:** 10.3390/nano10020244

**Published:** 2020-01-30

**Authors:** Renjing Huang, Yuan Cheng, Yichen Ji, Raymond J. Gorte

**Affiliations:** 1 Chemical & Biomolecular Engineering, University of Pennsylvania, Philadelphia, PA 19104, USA; hrenjing@seas.upenn.edu (R.H.); yuanc@seas.upenn.edu (Y.C.); yichenji@seas.upenn.edu (Y.J.); 2 Department of Materials Science and Engineering, University of Pennsylvania, Philadelphia, PA 19104, USA; 3 Catalysis Center for Energy Innovation, Newark, DE 19716, USA

**Keywords:** ethane dehydrogenation, cobalt, silica, atomic layer deposition (ALD), Co/SiO_2_, oxidative dehydrogenation, single-site catalysts

## Abstract

Unlike Co clusters, isolated Co atoms have been shown to be selective for catalytic dehydrogenation of ethane to ethylene; however, preparation of isolated Co sites requires special preparation procedures. Here, we demonstrate that Atomic Layer Deposition (ALD) of tris(2,2,6,6-tetramethyl-3,5-heptanedionato)cobalt(III) (Co(TMHD)_3_) on silica and other supports is effective in producing these isolated species. Silica-supported catalysts prepared with one ALD cycle showed ethylene selectivities greater than 96% at 923 K and were stable when CO_2_ was co-fed with the ethane. Co catalysts prepared by impregnation formed clusters that were significantly less active, selective, and stable. Rates and selectivities also decreased for catalysts with multiple ALD cycles. Isolated Co catalysts prepared on Al_2_O_3_ and MgAl_2_O_4_ showed reasonable selectivity for ethane dehydrogenation but were not as effective as their silica counterpart.

## 1. Introduction

Ethylene is one of the most important building blocks for the chemical industry and is widely used as a feedstock for producing polymers, ethylene oxide, and other derivatives [1]. Most ethylene has been produced by steam cracking of light naphtha or as a by-product of fluidized catalytic cracking of crude oil. More recently, steam cracking of ethane has taken on increased importance due to the abundance of ethane from shale gas [2]; however, lower-temperature dehydrogenation of ethane would be desirable for energy efficiency and reduced NO*_x_* emissions [3,4,5].

High temperatures are required in order to achieve high equilibrium conversions by direct catalytic dehydrogenation of ethane to H_2_ and ethylene. Oxidative dehydrogenation (ODH) eliminates the equilibrium requirement by consuming the H_2_ to form water, while simultaneously generating the heat required to raise the temperature of the reactants. The mostly extensively studied catalysts for ODH of ethane are chromium-based [6,7,8] and gallium-based [9,10] materials. In addition to other issues, the toxicity of CrO*_x_* and the severe coking behavior of GaO*_x_*-containing materials have prevented their wide-scale application. Other metal oxides, including those of V [11], Mo [11], and Co [12], have also been investigated and show some potential. Over-oxidation of the ethylene product is usually an issue in ODH with strong oxidants, and it would be particularly attractive if one could use a “soft” oxidant, such as readily available CO_2_ [13].

There have been several recent reports that cobalt-based catalysts can be active and selective for ODH of ethane using CO_2_ [12,14]. Of particular interest, Koirala et al. [12] demonstrated that flame-synthesis of CoO*_x_*/SiO_2_ produced a catalyst capable of selectivities above 85%. Their observation that selectivities increased with decreasing Co content suggested that the selective sites are associated with isolated Co. Spectroscopic data implied that the active species was Co^2+^ in a tetrahedral environment, strongly bound to the silica. Others have also shown that isolated Co can exhibit selective C–H activation of alkanes [15,16,17]. For example, Hu et al. used electrostatic adsorption of monomeric hexamminecobalt (III) with low metal loadings to produce silica-supported catalysts with isolated Co [15]. However, preparation of isolated Co species remains challenging; and conventional preparation by impregnation usually results in catalysts that have metal atoms in a variety of coordination environments [16].

In the present work, we will demonstrate that isolated Co sites on silica and other supports can be prepared easily by atomic layer deposition (ALD). The basic idea behind ALD is that a precursor reactant is first allowed to react with a surface in a self-terminating way. After the excess precursor has been purged, the adsorbed precursor is oxidized in a separate step. By choosing the correct conditions, conformal films can be deposited on either flat or porous surfaces, as described in several reviews [18,19]. For the present application, it is important to recognize that a single deposition cycle of Co precursors with large ligands results in spatially isolated Co atoms that retain that site isolation after removal of the ligands. For example, previous work has shown that the growth rate for ALD with tris(2,2,6,6-tetramethyl-3,5-heptanedionato)cobalt(III) (Co(TMHD)_3_) is 5 × 10^17^ Co/m^2^ [20,21], a coverage that is roughly a factor of ten lower than that which would be expected for Co in a monolayer of CoO*_x_*. This low coverage per cycle, which is almost certainly due to steric crowding by the large TMHD ligands, causes the Co ions to be spatially isolated. The resulting catalysts exhibit exceptional activity, selectivity, and stability for dehydrogenation of ethane. A comparison of Co^2+^ on silica, alumina, and MgAl_2_O_4_ shows that the silica-supported catalysts are superior but reasonable performance could be achieved with isolated Co^2+^ on the other supports as well.

## 2. Materials and Methods

### 2.1. Catalyst Preparation

SiO_2_, Al_2_O_3_, and MgAl_2_O_4_ were used as the supports in this work. The SiO_2_ (Fuji Silysia Chemical G-6, Nagoya, Japan, 10 μm) was calcined in air at 973 K for 1 h. The Al_2_O_3_ used in this study was obtained as γ-Al_2_O_3_ (Strem Chemicals, Newburyport, MA, USA) but was then stabilized by calcination in air at 1173 K for 24 h. The MgAl_2_O_4_ was prepared in our laboratory by co-precipitation and the synthesis has been described in previous publications [22]. It was also calcined in air at 1173 K for 12 h to stabilize its surface area. All support powders were pressed into thin wafers and then broken into small pieces before further use.

The cobalt was deposited onto different supports by ALD using a home-built, static system that has been described in detail previously [23,24]. The ALD precursor was Co(TMHD)_3_ (Strem Chemicals). During the deposition process, approximately 0.7 g of support material were evacuated and exposed to 5 Torr of the precursor vapor at 523 K for 5 min, after which the samples were evacuated to remove excess precursors and then oxidized in a muffle furnace at 773 K for 7 min to fully remove the ligands. Samples with n ALD cycles on a particular support are referred to here as *nCo-support*.

A silica-supported sample was also prepared by conventional incipient wetness, using aqueous solutions of Cobalt(II) nitrate hexahydrate (98%, Sigma-Aldrich, St. Louis, MO, USA). To minimize CoO*_x_* cluster size, this sample was prepared with 1-wt% Co on SiO_2_. After impregnation, it was calcined for 3 h at 873 K and is referred to as *impCo-SiO_2_*.

### 2.2. Catalyst Characterization

X-ray diffraction (XRD) patterns were measured on a MiniFlex diffractometer (Rigaku, Tokyo, Japan) equipped with a Cu Kα source (*λ* = 154.05 pm). Co loadings were initially determined by weight changes following ALD but then confirmed by Inductively-Coupled Plasma, Optical Emission Spectrometry (ICP-OES, Spectro Genesis, Kleve, Germany). Specific surface areas were obtained by the Brunauer–Emmett–Teller (BET; home-built device) method using N_2_ at 78 K. UV–Vis, diffuse-reflectance spectra were recorded on a Cary 5000 UV–Vis–NIR spectrophotometer equipped with a Praying Mantis diffuse reflectance accessory (Harrick Scientific Products, Pleasantville, NY, USA). The spectra were measured against a Spectralon background. A thermogravimetric analyzer (Q600, TA Instruments, New Castle, DE, USA) was used to quantify the amount of coke in the spent catalyst after 24 h on stream. For these measurements, the samples were first held at 373 K for 1 h in flowing, dry N_2_ before ramping the temperature in flowing air to 1173 K at a rate of 10 K/min. Temperature programmed desorption (TPD) measurements were performed in a high-vacuum system (10^−7^ torr), using 50-mg samples and a quadrupole mass spectrometer (RGA100,Stanford Research Systems, Sunnyvale, CA, USA) for product detection [25]. The heating rate in these measurements was again 10 K/min.

### 2.3. Catalyst Testing

The dehydrogenation reactions were conducted in a 1/2-inch, quartz, tubular flow reactor. Gas flow was controlled using simple rotameters with metering valves (FL3600-3800, Omega Engineering, Norwalk, CT, USA). Products were detected by on-line gas chromatography (Model 310, Buck Scientific, city, country) with a Haysep Q column and a thermal conductivity detector using Helium (He, 99.999%, Airgas, Allentown, PA, USA) as the carrier gas. In a typical run, 0.5 g of catalyst was loaded in the center of the tube between two quartz-wool plugs. The catalysts were heated to the desired reaction temperature at 10 K/min in He flow before introducing the reaction mixtures. The reactant gases were ethane (C_2_H_6_, 99.99%, Airgas) and carbon dioxide (CO_2_, 99.99%, Airgas). The total flow rate of the reactant gases and He was maintained at 30 mL/min throughout the study. To increase the residence time, we either increased the catalyst loading or reduced the total flow rate. In all cases, the carbon balances were greater than 98% for reactions when CO_2_ and ethane were co-fed and 96% in the absence of CO_2_.

## 3. Results

### 3.1. Sample Characterization

A list of all the samples used in this study is reported in Table 1, together with their metal loadings and BET surface areas. The specific Co coverages in the table were calculated from the Co weight loading and the BET surface area, assuming the Co was present as CoO. On the SiO_2_ support, catalysts were prepared with between one and ten ALD cycles of Co(TMHD)_3_. As shown in Table 1, the Co loading increased linearly with the number of cycles, as expected; however, the growth rate, 2.5 × 10^17^ Co/m^2^-cycle, was about half that reported previously for deposition of Co with this precursor on Al_2_O_3_ and MgAl_2_O_4_ [20,21]. Since only one ALD cycle was deposited onto the Al_2_O_3_ and MgAl_2_O_4_ supports in the present study, there was significant uncertainty in the growth rates for these samples; but the values, 8 × 10^17^ Co/m^2^-cycle, were more similar to what has been reported previously [20,21]. We suggest that the lower growth rate on SiO_2_ was due to the fact that this support had a much higher surface area than the supports used in earlier work and therefore had smaller pores. It is likely that the Co precursor with large TMHD ligands was unable to access smaller pores. However, we cannot rule out the possibility that the silica simply had a lower concentration of reactive sites [26].

It is useful to notice that the coverage of 2.7 × 10^17^ Co/m^2^ would imply that each Co occupies an area of 4 nm^2^, which would indicate that the metal atoms are separated by a distance of roughly 2 nm. Even after 10 ALD cycles, the average distance between Co atoms is expected to be greater than 0.6 nm. Finally, the decrease in BET surface area with the number of ALD cycles in the SiO_2_-supported samples is mainly due to the increased mass of the samples, combined with a small change in pore size [27].

**Figure 1:**
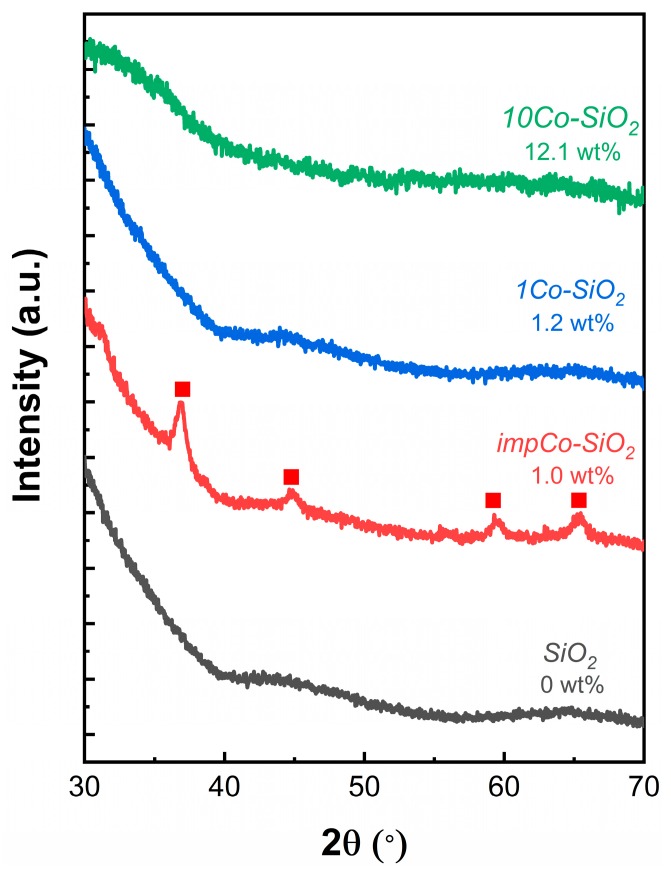
XRD patterns of *SiO_2_*, *impCo-SiO_2_*, *1Co-SiO_2_*, and *10Co-SiO_2_*. Characteristic peaks associated with Co_3_O_4_ are marked as (■).

XRD patterns of the *SiO_2_*, *1Co-SiO_2_*, *10Co-SiO_2_*, and *impCo-SiO_2_* samples, shown in Figure 1, demonstrate that the structure of the ALD-prepared samples differed significantly from that of the sample prepared by impregnation. Each of these samples was calcined above 773 K but only the *impCo-SiO_2_* sample exhibited features at 36, 44, 59, and 65 degrees 2θ. These peaks correspond to Co_3_O_4_ and demonstrate that, in the absence of very specialized procedures, such as the electrostatic adsorption procedure used by Hu et al. [15,28], impregnation leads to formation of metal clusters, even at relatively low Co loadings. From the XRD peak width and Scherrer’s equation, the Co_3_O_4_ crystallite size for *impCo-SiO_2_* was estimated to be 11 nm. By contrast, the samples prepared by ALD showed no observable XRD peaks for cobalt oxides, even at Co loadings ten times higher.

**Figure 2:**
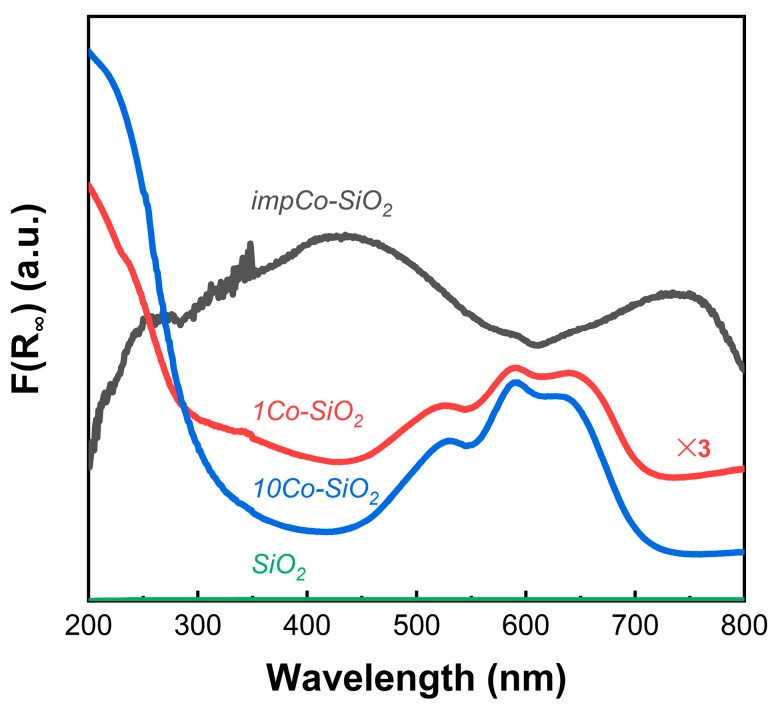
UV–Vis diffuse reflectance spectra of *SiO_2_*, *1Co-SiO_2_*, *10Co-SiO_2_,* and *impCo-SiO_2_*.

The difference in the structure of the materials prepared by ALD and by impregnation was further shown by the UV–Vis spectra in Figure 2. Pure SiO_2_ did not adsorb in the UV–Vis range that is shown here and thus did not exhibit any features. The *impCo-SiO_2_* sample was visibly darker and exhibited broad absorption bands between 300 and 600 nm and between 650 and 800 nm. These bands were consistent with that expected for Co_3_O_4_ [29]. However, the *1Co-SiO_2_* and *10Co-SiO_2_* were both light blue in color and exhibited absorption bands centered at 530, 590, and 640 nm. These spectra imply that the Co is present as isolated Co^2+^ in a tetrahedral environment [17,28,30]. As expected, the intensity of the bands was stronger with *10Co-SiO_2_* compared to *1Co-SiO_2_* due to the increased metal loading but the features remained the same. It is also noteworthy that these spectra are the same as those reported for the flame-prepared samples in an earlier study [12,28].

**Figure 3:**
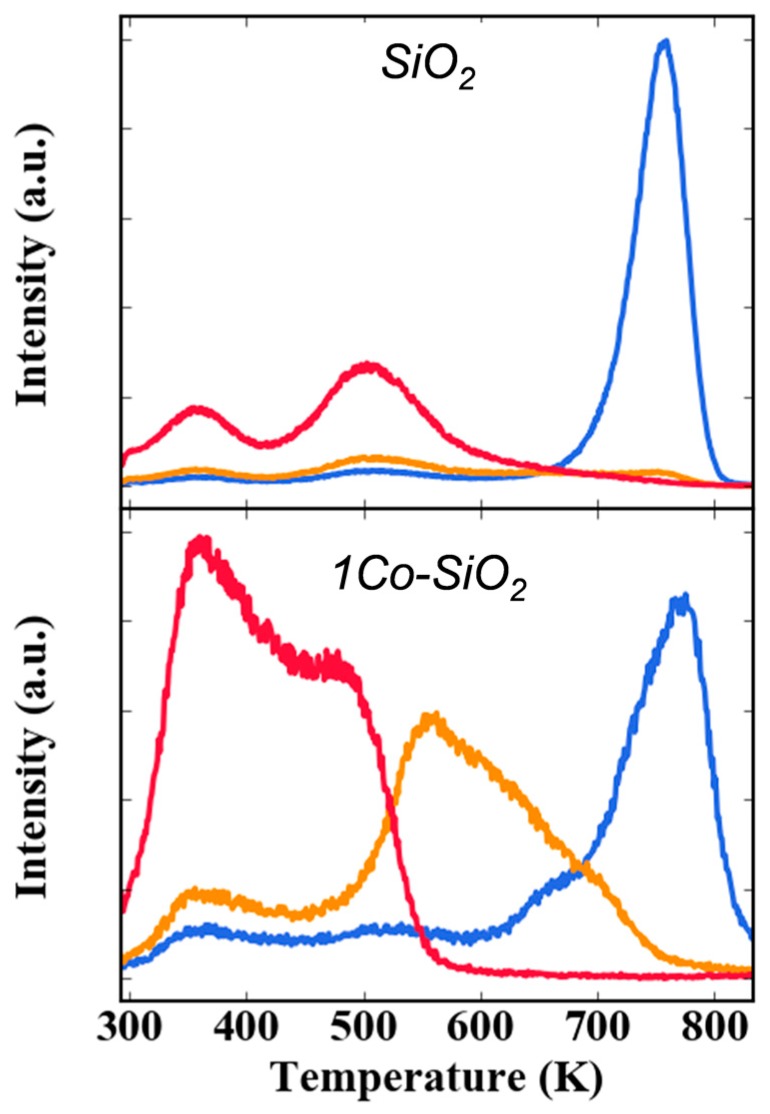
Temperature programmed desorption (TPD) of 2-propanol following room-temperature adsorption on *SiO_2_* and *1Co-SiO_2_*. The desorption features represent: 2-propanol (red), acetone (orange) and propene (blue).

The adsorption properties of the Co added by ALD were probed in TPD studies with 2-propanol, with results shown for *SiO_2_* and *1Co-SiO_2_* in Figure 3. 2-Propanol adsorbs on silica via hydrogen bonding with surface silanols [31]. Some of the alcohol desorbed as 2-propanol below 550 K but a significant fraction desorbed as propene between 700 and 800 K. The propene was produced by the decomposition of the silyl/iso-propyl ether that formed when the alcohol reacted with silanols. The water formed in this reaction desorbed at lower temperatures, over a broad temperature range, so that the silyl/iso-propyl ether that remained at higher temperatures could not recombine back to the alcohol. While these same silica-related features were observed on the *1Co-SiO_2_* sample, the Co-containing sample also exhibited acetone formation between 500 and 650 K. The formation of acetone is a clear indication of dehydrogenation activity.

### 3.2. Catalytic Performance

#### 3.2.1. Comparison of 1Co-SiO_2_ and impCo-SiO_2_

**Figure 4:**
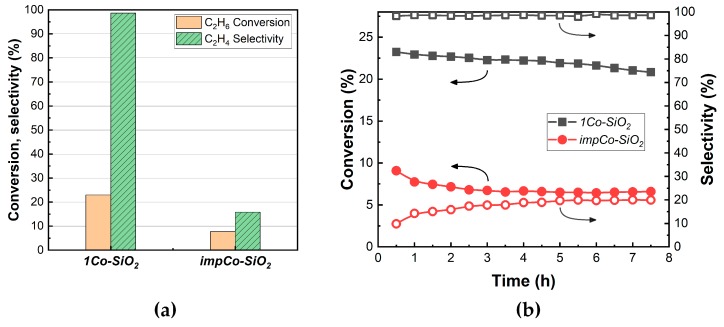
Comparison of *1Co-SiO_2_* and *impCo-SiO_2_* for the reaction of CO_2_ and C_2_H_6_. Reaction conditions were: T = 923 K; CO_2_:C_2_H_6_:He = 1:1:4; space time = 1 h·g_cat_·mL^−1^. (**a**) C_2_H_6_ conversions and selectivity after 1 h. (**b**) Time-on-stream curves for C_2_H_6_ conversions and selectivities to C_2_H_4_ for *1Co-SiO_2_* (■, □)) and *impCo-SiO_2_* (●, ○).

To understand how the catalytic properties of isolated Co sites differ from that of CoO*_x_* clusters, we examined the reaction of ethane and CO_2_ on the *1Co-SiO_2_* and *impCo-SiO_2_* samples at 923 K, with data shown in Figure 4. These experiments were performed on the oxidized samples but prereduction of the *1Co-SiO_2_* sample at 973 K in H_2_ had no effect on either the conversion or the selectivity. As shown in Figure 4a, using the same amounts of catalyst and a CO_2_:C_2_H_6_ ratio of one, the conversion on *1Co-SiO_2_* was approximately three times higher than on *impCo-SiO_2_*, 22% versus 7%. More importantly, the selectivity on *1Co-SiO_2_* was 99% to ethylene, compared to a value of only 16% on *impCo-SiO_2_*. The *impCo-SiO_2_* acted primarily as a reforming catalyst, producing CO and H_2_. The *1Co-SiO_2_* sample was also relatively stable for a period of at least 7 h, Figure 4b. Since the results on *impCo-SiO_2_* show that clusters were not selective for ethylene, the fact that the selectivity remained high on *1Co-SiO_2_* implies that the isolated Co species did not sinter to form clusters under these conditions. Finally, it is interesting to note that the selectivities that we observed on *1Co-SiO_2_* were actually somewhat higher than the dilute-Co catalysts that were flame-prepared by Koirala et al. [28]. While the conditions of our experiments were clearly different, this result suggests that preparation of the catalyst by ALD may be more effective in producing isolated Co compared to the flame-preparation method.

#### 3.2.2. Influence of CO_2_ Partial Pressure

**Figure 5:**
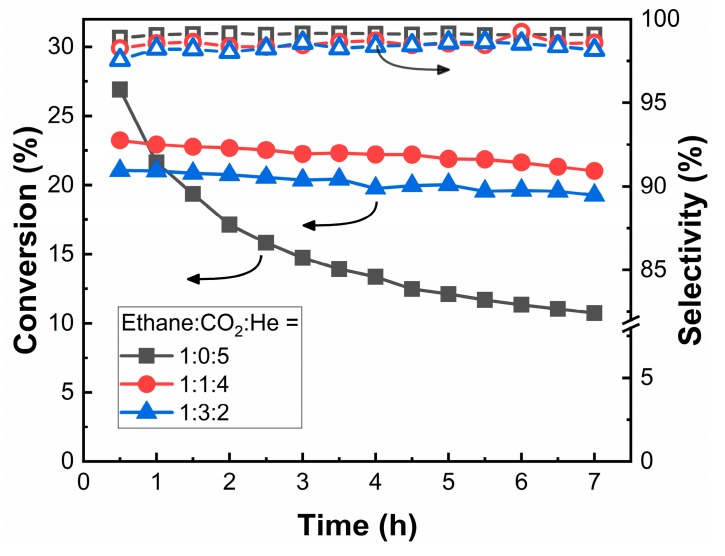
Effect of CO_2_ on ethane conversion and selectivity to ethylene over *1Co-SiO_2_*: C_2_H_6_:CO_2_:He = 1:0:5 (■, □); =1:1:4 (●, ○); and = 1:3:2 (▲, △). Reaction conditions: T = 923 K; space time = 1 h·g_cat_·mL^−1^.

In the reaction of ethane with CO_2_, it has been proposed that the role of CO_2_ can be either to push the equilibrium towards ethylene production by consuming H_2_ via reverse water gas shift (RWGS) or to remove coke via reverse Boudouard reaction [1,2,32]. To determine which process was more important for the present system, we examined the conversion and stability of the *1Co-SiO_2_* catalyst for different CO_2_:C_2_H_6_ ratios, with results shown in Figure 5. Measurements were performed as a function of time at a fixed mole fraction of C_2_H_6_ of 0.167 and a fixed space time (30 mL/min total flow rate with 0.5-g catalyst). In all cases, the selectivity to ethylene was greater than 97%, demonstrating that the presence of CO_2_ did not affect selectivity. In the absence of CO_2_, the conversions were initially higher than what we measured in the presence of CO_2_, implying that equilibrium considerations are not important. This conclusion is further supported by the fact that the equilibrium conversion of ethane to ethylene and H_2_ at this temperature would be 69%. Furthermore, even with a CO_2_:C_2_H_6_ ratio of 3, only about 5% of the H_2_ produced in the reaction was consumed by RWGS. However, in the absence of CO_2_, the conversion declined significantly with time. For CO_2_:C_2_H_6_ ratios of both 1 and 3, the conversions were stable. The effect of changing the CO_2_:C_2_H_6_ ratio was modest so long as some CO_2_ was present in the reaction mixture. The conversions decreased, but only slightly, when the CO_2_:C_2_H_6_ ratio increased from 1 to 3.

#### 3.2.3. Stability and Regeneration of 1Co-SiO_2_

To gain further insights into the reasons for deactivation of *1Co-SiO_2_* in the absence of CO_2_, we performed additional, 24-h tests of the sample, with and without CO_2_. We also measured the carbon contents of the sample after running the reaction for this amount of time and attempted to regenerate the catalyst by oxidation. The results of these experiments are shown in Table 2. In agreement with Figure 5, the conversion of ethane in the absence of CO_2_ fell to 7.2% after 24 h while the conversion dropped only modestly, from 23.2% to 18.1%, when CO_2_ was added to the feed. The activity of the catalyst could be largely restored by oxidation at 923 K in flowing air, implying that deactivation was not due to some structural change in the catalyst. The carbon contents after 24 h of reaction were 3.7-wt% in the absence of CO_2_ and 1.0-wt% when the feed contained CO_2_. Since metallic Co nanoparticles can form large amounts of carbon due whisker formation [33,34], both of these values were relatively small, indicating that whisker formation did not occur; however, on an atomic basis, the C:Co ratio corresponding to 3.7-wt% C was greater than 20, so that this amount of carbon was sufficient to cover the active component.

#### 3.2.4. Effects of Reaction Temperature and Space Velocity

**Figure 6:**
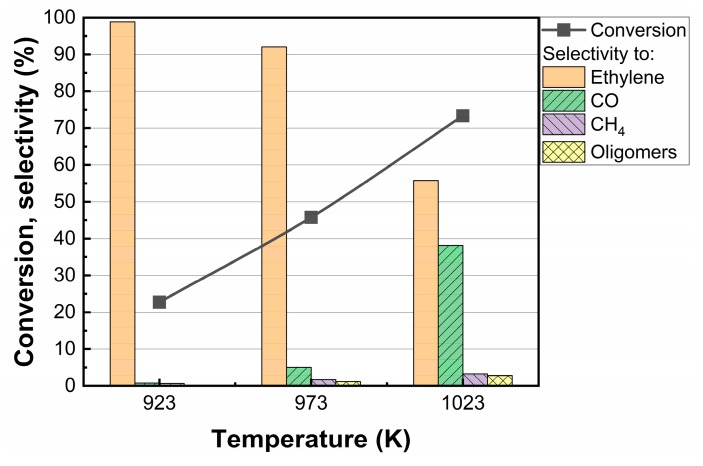
Effect of temperature on the conversion and selectivity for ethane dehydrogenation over *1Co-SiO_2_*. Data were collected at a time-on-stream of 1 h. Reaction conditions: T = 923 K; CO_2_:C_2_H_6_:He = 1:1:4; space time = 1 h·g_cat_·mL^−1^.

The effect of reaction temperature on the *1Co-SiO_2_* sample was investigated between 923 and 1023 K, with results reported in Figure 6. While conversion increased with temperature, the selectivity decreased dramatically. At higher reaction temperatures, we observed evidence for both gas-phase, free-radical reactions from the presence of methane and higher-molecular weight products and dry reforming from the presence of CO in the products.

**Figure 7:**
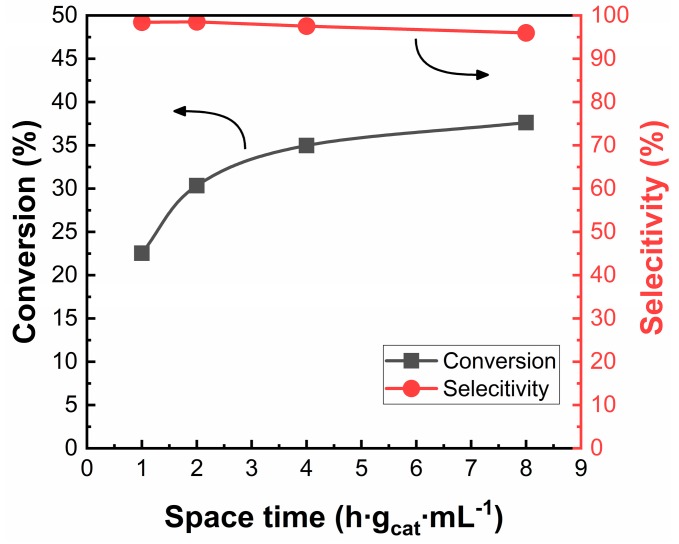
Effect of space time on catalytic performance for ethane dehydrogenation over *1Co-SiO_2_*. Data were collected at a time-on-stream of 2 h. Reaction conditions: T = 923 K; CO_2_:C_2_H_6_:He = 1:1:4.

The effect of varying the space time on the *1Co-SiO_2_* sample is shown in Figure 7. As expected, the conversion increased with increasing space time. What is noteworthy is that the selectivity to ethylene remained high, greater than 96%, at conversions approaching 40%. Ethylene did not appear to be reactive on this catalyst under these conditions.

#### 3.2.5. Effect of the Number of ALD Cycles on SiO_2_

**Figure 8:**
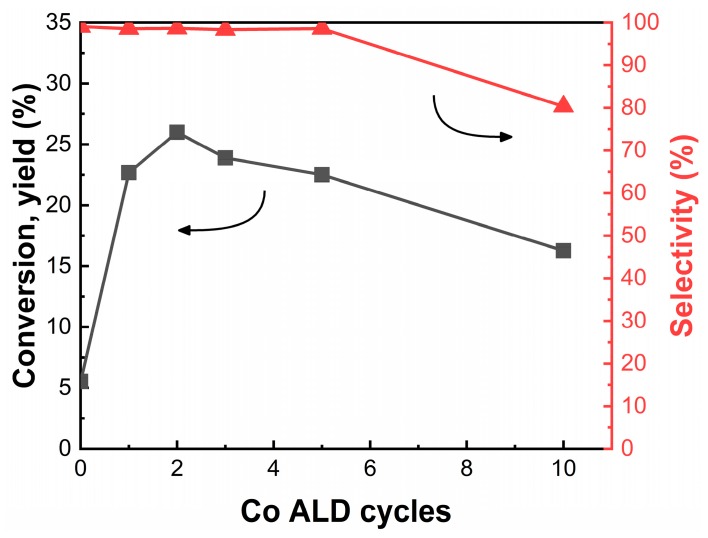
Conversion (■) and selectivity (▲) on SiO_2_-supported catalysts as a function of the number of Co ALD cycles. Data were collected at a time-on-stream of 2 h. Reaction conditions: T = 923 K; CO_2_:C_2_H_6_:He = 1:1:4; space time = 1 h·g_cat_·mL^−1^.

It is expected that higher Co loadings should lead to higher rates if isolated Co sites can be maintained. Since the XRD and UV–Vis results in Figure 1 and Figure 2 suggest that the Co still exists as isolated sites following multiple ALD cycles, we examined the conversion and selectivity of SiO_2_-supported samples as a function of the number of ALD cycles, with results shown in Figure 8. The conversion did increase slightly after the second ALD cycle but then declined monotonically. Selectivity remained high after as many as five ALD cycles but then decreased dramatically after 10 ALD cycles. We suggest that the most likely explanation for the decrease in rates with increasing ALD cycles is that only isolated Co sites are active. Additional ALD cycles create sites in which Co sites are paired, so that adding more Co lowers the rates. After 10 ALD cycles, some of the Co is present as clusters, which are active for non-selective reactions.

#### 3.2.6. Isolated Co on Other Supports

**Figure 9:**
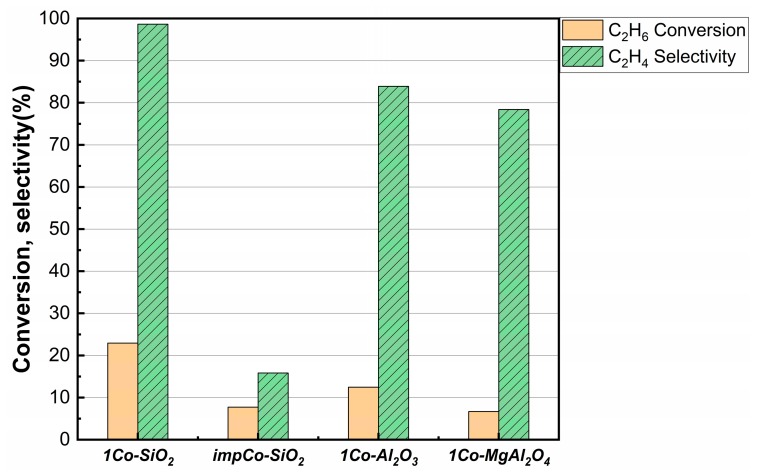
Conversions and selectivities for *1Co-SiO_2_*, *impCo-SiO_2_*, *1Co-Al_2_O_3_*, and *1Co-MgAl_2_O_4_*. Data were collected at a time-on-stream of 1 h. Reaction conditions: T = 923 K; CO_2_:C_2_H_6_:He = 1:1:4; space time = 1 h·g_cat_·mL^−1^.

Unlike the flame-synthesis [12] and electrostatic-adsorption [15] methods for preparing isolated Co sites, addition of Co by ALD is expected to provide isolated sites independent of the support. Therefore, to determine the effect of different supports on the reaction of ethane, samples were prepared with one ALD cycle of Co(TMHD)_3_ onto Al_2_O_3_ and MgAl_2_O_4_. Although Co is expected to react with Al_2_O_3_ to form CoAl_2_O_4_ at higher temperatures [20], the presence of MgO should provide some stability [21]. Figure 9 compares the conversions and selectivities at a time-on-stream of 1 h for *1Co-SiO_2_*, *impCo-SiO_2_*, *1Co-Al_2_O_3_*, and *1Co-MgAl_2_O_4_*. The *1Co-Al_2_O_3_* and *1Co-MgAl_2_O_4_* samples were less active and selective compared to *1Co-SiO_2_* but both catalysts were significantly better than *impCo-SiO_2_*. The lower activity of *1Co-Al_2_O_3_* and *1Co-MgAl_2_O_4_* compared to *1Co-SiO_2_* could be explained by Co incorporation into the bulk of the Al_2_O_3_ and MgAl_2_O_4_ supports. While reaction of Co with the Al_2_O_3_ and MgAl_2_O_4_ supports cannot explain the higher selectivity of the *1Co-SiO_2_* catalyst, these catalysts remained significantly better than the *impCo-SiO_2_*, suggesting clusters were not formed.

## 4. Discussion

In this work, we have demonstrated that isolated-site, Co catalysts can be synthesized readily using ALD with large precursor molecules. While other methods for preparing single-site catalysts have been demonstrated [12,15], the utility of ALD is that the conditions required to make the materials appear to be independent of support. One ALD cycle also appears to provide a nearly optimal metal loading, so that the effect of loading does not need to be explored. We should also note that preparation of materials using one ALD cycle can be accomplished in a simple, high-temperature adsorption system and does not require the expensive equipment that is more commonly used when rapid cycling is required [23]. Furthermore, since the oxidation step can be carried out at higher temperatures, possibly in a separate system, the precursor does not need to be a specialized compound designed to be easily oxidized at the deposition temperature. Any compound that remains stable to temperatures at which it has a vapor pressure can be used.

Isolated-site catalysts based on Zn have been successfully prepared by ALD in earlier studies [35], but the approach does not appear to have been successful in preparing single-atom, precious-metal catalysts [21,36,37]. A key factor in being able to achieve single-site catalysts is the requirement that the metal atoms be immobilized on the surface. Success in the case of Zn and Co is likely due to the fact that these atomic species remain in an oxidized form, bonded in some way to the support. This is likely the case also for Co. While bulk Co would be reduced to its zero-valence state at the low O_2_ fugacities associated with ethane dehydrogenation, isolated Co atoms are likely much harder to reduce [38]. Furthermore, it has recently been demonstrated that interactions with the support can shift equilibrium constants to make the metals even more difficult to reduce [38]. By contrast, precious metals are always much easier to reduce. In the case of single-atom, precious-metal catalysts, the support almost always plays a key role [39,40].

Isolated-site Co catalysts are especially intriguing, given their high selectivity for dehydrogenation of ethane and remarkable tolerance against coking. The fact that we were able to achieve relatively high conversions (>25%) with almost 100% selectivity (99%) is very encouraging. Whether silica is playing a role as “ligand” for the supported Co is uncertain. While rates for isolated Co appear to be lower for Co on Al_2_O_3_ and MgAl_2_O_4_, this could be associated with formation of CoAl_2_O_4_. The Co/Al_2_O_3_ and Co/MgAl_2_O_4_ catalysts were still reasonably selective. It will be interesting to investigate the effects of other supports that may be less reactive.

Obviously, there is still much to learn about the nature and properties of the catalytic sites in single-site cobalt on silica. We believe the controlled synthesis of ALD could open the possibility for understanding and producing highly active and selective Co-based catalyst for various applications.

## 5. Conclusions

We have successfully prepared isolated-site, Co catalysts on SiO_2_, Al_2_O_3_, and MgAl_2_O_4_ supports using ALD. The SiO_2_-supported Co catalyst exhibited dramatically improved conversion and selectivity for ethane dehydrogenation compared to a catalyst prepared by the conventional impregnation. ALD provides a simple method for preparing these isolated-site catalysts.

## Figures and Tables

**Table 1:** Samples used in this study, together with their Brunauer–Emmett–Teller (BET) surface areas and Co loadings. *nCo-MO_x_* correspond to samples prepared by Atomic Layer Deposition (ALD), with n being the number of ALD cycles.

Sample	BET S.A (m^2^/g)	Metal Loading (wt%)	Cobalt Coverages (Co/m^2^)
*SiO_2_*	472	0	0
*1Co-SiO_2_*	457	1.2	2.7 × 10^17^
*2Co-SiO_2_*	445	2.3	5.3 × 10^17^
*3Co-SiO_2_*	438	3.5	8.2 × 10^17^
*5Co-SiO_2_*	419	5.5	1.3 × 10^18^
*10Co-SiO_2_*	343	12.1	3.6 × 10^18^
*impCo-SiO_2_*	453	1.0	2.3 × 10^17^
*1Co-Al_2_O_3_*	102	0.9	9.0 × 10^17^
*1Co-MgAl_2_O_4_*	136	1.0	7.5 × 10^17^

**Table 2:** The conversions and selectivities for ethane dehydrogenation over *1Co-SiO_2_*, initially and after 24 h, with and without CO_2_ in the feed. Reaction conditions: T = 923 K; space time = 1 h·g_cat_·mL^−1^. Regeneration was performed in flowing air at 923 K for 1 h.

Feed	Conversion (Selectivity) %			Amount of Coke
C_2_H_6_:CO_2_:He	0.5 h	24 h	After Regen.	
1:0:5	26.9 (98.8)	7.2 (98.8)	21.7 (98.8)	3.7 wt%
1:1:4	23.2 (98.3)	18.1 (98.0)	17.0 (97.8)	1.0 wt%
